# Supplementary material for: Cardioprotective effects of early versus late initiated antiretroviral treatment in adolescents with perinatal HIV-1 infection
Source: Sci Rep. 2024 Jun 20;14:14234. doi: 10.1038/s41598-024-65119-9 (PMC11189904; doi:10.1038/s41598-024-65119-9)
Supplement: Supplementary file 1 — Supplementary Table S1. [file 41598_2024_65119_MOESM1_ESM.docx]

**Cardioprotective effects of early versus late initiated antiretroviral treatment in adolescents with perinatal HIV-1 infection.**

Itai M Magodoro^1^, Carlos E Guerrero-Chalela^2^, Brian Claggett^3, 4^, Stephen Jermy^5^, Petronella Samuels^5^, Landon Myer^6^, Heather Zar^7^, Jennifer Jao^8, 9^, Mpiko Ntsekhe^1^, Mark J Siedner^10, 11^, Ntobeko AB Ntusi^1, 5, 12, 13^*

1. Department of Medicine, University of Cape Town, Cape Town, South Africa
2. Fundación Cardioinfantil Instituto de Cardiología, Bogotá D.C., Colombia
3. Cardiology Division, Brigham and Women’s Hospital, Boston, MA, USA
4. Harvard Medical School, Boston, MA, USA
5. Cape Universities Body Imaging Centre, University of Cape Town, Cape Town, South Africa
6. Division of Epidemiology and Biostatistics, School of Public Health and Family Medicine, University of Cape Town, Cape Town, South Africa
7. Department of Pediatrics and Child Health, and SA-MRC Unit on Child and Adolescent Health, University of Cape Town, Cape Town, South Africa
8. Department of Pediatrics, Division of Pediatric Infectious Diseases, Northwestern University Feinberg School of Medicine, Chicago, IL, USA
9. Department of Internal Medicine, Division of Adult Infectious Diseases, Northwestern University Feinberg School of Medicine, Chicago, IL, USA
10. Center for Global Health, Massachusetts General Hospital, Boston, MA, USA
11. Africa Health Research Institute, KwaZulu-Natal, South Africa
12. South African Medical Research Council Extramural Unit on Noncommunicable Diseases and Infectious Diseases, Cape Town, South Africa
13. ARUA/Guild Cluster of Research Excellence on Noncommunicable Diseases and Associated Multimorbidity

**Supplementary Table S1. Cardiac magnetic resonance (CMR) sequence parameters.**

| **Technique** | **Cine imaging** | **T1 mapping** | **T2 mapping** | **Late gadolinium enhancement** |
| --- | --- | --- | --- | --- |
| Sequence | Segmented balanced steady state free precession (bSSFP) | Modified Look-Locker Inversion-Recovery (MOLLI) | T2-prepared bSSFP | T1- weighted segmented gradient echo inversion recovery |
| FOV read (mm) | 360 | 360 | 360 | 360 |
| FOV phase (%) | 75 | 75 | 75 | 75 |
| TR (ms) | 45 | 270 | 204 | 874 |
| TE (ms) | 1.67 | 1.12 | 1.32 | 1.95 |
| Flip angle (°) | 40 | 35 | 12 | 20 |
| Voxel size (mm^3^) | 1.73 x 1.73 x 8.0 | 1.40 x 1.40 x 8.0 | 1.88 x 1.88 x 8.0 | 1.41 x 1.41 x 8.0 |
| Comments | 25 phases per RR | 5(3)3 | 0; 25; 55 ms | Magnevist (0.2 ml/kg) |

FOV = field of view; TR = repetition time; TE = echo time.
